# Temporomandibular Joint Osteoarthritis: Regenerative Treatment by a Stem Cell Containing Advanced Therapy Medicinal Product (ATMP)—An In Vivo Animal Trial

**DOI:** 10.3390/ijms22010443

**Published:** 2021-01-05

**Authors:** Robert Köhnke, Marcus Oliver Ahlers, Moritz Alexander Birkelbach, Florian Ewald, Michael Krueger, Imke Fiedler, Björn Busse, Max Heiland, Tobias Vollkommer, Martin Gosau, Ralf Smeets, Rico Rutkowski

**Affiliations:** 1 Department of Oral and Maxillofacial Surgery, University Medical Center Hamburg-Eppendorf, 20246 Hamburg, Germany; r.koehnke@uke.de (R.K.); m.birkelbach@uke.de (M.A.B.); t.vollkommer@uke.de (T.V.); m.gosau@uke.de (M.G.); r.smeets@uke.de (R.S.); 2 Department of Prosthetic Dentistry School of Dental Medicine, University Medical Center Hamburg-Eppendorf, 20246 Hamburg, Germany; ahlers@uke.de; 3 CMD-Center Hamburg-Eppendorf, 20251 Hamburg, Germany; 4 Department of General, Visceral and Thoracic Surgery, University Medical Center Hamburg Eppendorf, 20246 Hamburg, Germany; f.ewald@uke.de; 5 OXACELL^®^ AG, 14469 Potsdam, Germany; dr_krueger@aol.com; 6 Department of Osteology and Biomechanics, University Medical Center Hamburg-Eppendorf, 20246 Hamburg, Germany; i.fiedler@uke.de (I.F.); b.busse@uke.de (B.B.); 7 Charité–Universitätsmedizin Berlin, Corporate Member of Freie Universität Berlin, Humboldt-Universität zu Berlin, and Berlin Institute of Health, Department of Oral and Maxillofacial Surgery, 14197 Berlin, Germany; max.heiland@charite.de; 8 Department of Oral and Maxillofacial Surgery, Division of Regenerative Orofacial Medicine, University Medical Center Hamburg-Eppendorf, 20246 Hamburg, Germany

**Keywords:** osteoarthritis, temporomandibular joint, regenerative therapy, stem cell therapy, regenerative medicine, TMJD

## Abstract

Temporomandibular joint osteoarthritis (TMJ-OA) is a chronic degenerative disease that is often characterized by progressive impairment of the temporomandibular functional unit. The aim of this randomized controlled animal trial was a comparative analysis regarding the chondroregenerative potency of intra-articular stem/stromal cell therapy. Four weeks after combined mechanical and biochemical osteoarthritis induction in 28 rabbits, therapy was initiated by a single intra-articular injection, randomized into the following groups: Group 1: AB Serum (ABS); Group 2: Hyaluronic acid (HA); Group 3: Mesenchymal stromal cells (STx.); Group 4: Mesenchymal stromal cells in hyaluronic acid (HA + STx.). After another 4 weeks, the animals were euthanized, followed by histological examination of the removed joints. The histological analysis showed a significant increase in cartilage thickness in the stromal cell treated groups (HA + STx. vs. ABS, *p* = 0.028; HA + ST.x vs. HA, *p* = 0.042; STx. vs. ABS, *p* = 0.036). Scanning electron microscopy detected a similar heterogeneity of mineralization and tissue porosity in the subchondral zone in all groups. The single intra-articular injection of a stem cell containing, GMP-compliant advanced therapy medicinal product for the treatment of iatrogen induced osteoarthritis of the temporomandibular joint shows a chondroregenerative effect.

## 1. Introduction

Temporomandibular joint disorders (TMJD) comprise a disease group with complex etiology (e.g., inflammatory, traumatic, genetic) [1]. While pain is often the leading and most distressing sign described by patients, the individual symptoms also include aspects such as joint noise, limited range of motion, impaired jaw function, malocclusion and deviation or deflection upon mouth opening [2,3]. TMJ-osteoarthritis (TMJ-OA) represents an important subtype of TMJD. In the Research Diagnostic Criteria for Temporo-Mandibular Disorders (RDC/TMD) it is classified in group 3. TMJ-OA can arise primarily or secondarily as a consequence of other subtypes (for example, disc displacement) and is characterized by progressive cartilage degeneration and subchondral bone sclerosis [4,5].

The treatment principles of TMJ-OA focus on reducing joint pain, increasing joint function and preventing progressive joint destruction. Further aims are the prevention of a disease-related reduction of quality of life and overall morbidity. In general, the treatment principles comprise non-invasive, minimally invasive and invasive procedures. The most common non-invasive modalities include occlusal splints to (re-)establish a balanced occlusion as well as a most stable and least joint-traumatizing condylar position, thus protecting the temporomandibular joint from involuntary overloading and also reducing muscular hyperactivity [6,7]. Furthermore, physiotherapy and electrophysical techniques (e.g., transcutaneous electrical nerve stimulation (TENS)) are classified as non-invasive procedures [8,9]. The primary aim of these non-invasive measures is local pain relief, suppression of inflammation and an increase in local perfusion. In the pharmacological field, analgesics (especially non-steroidal anti-inflammatory drugs (NSAIDs)) and indication-specific muscle relaxants are among the most frequently prescribed drugs [10,11]. Minimally invasive treatments include injection therapies, either into the masticatory muscles (Botulinum toxin type A (Botox)) or intra-articular (e.g., hyaluronic acid, corticosteroids, platelet rich plasma (PRP)) but also arthrocentesis (lavage) and arthroscopy, which enables the visualization and treatment of pathological intra-articular structures [12,13,14,15,16]. Invasive procedures, including open joint surgery, either aim to restore the temporomandibular joint or to replace it completely with autogenous or alloplastic material [17]. However, despite the broad spectrum of well-established treatment options, permanent recovery of the TMJ is rarely obtained. Hence it is not uncommon for therapy-refractory symptoms to occur with progressive impairment of the temporomandibular joint function [14].

Considering the increasing knowledge about the pathophysiological basics of TMJ-OA as well as recent advances in the understanding of stem cell biology and biomaterials, approaches in the field of tissue engineering and assisted tissue regeneration may present promising treatment alternatives. So far, the TMJ represents an enormous challenge for regenerative techniques due to the complex structural joint composition including bone, cartilage, ligaments, muscles and synovial membranes. Furthermore, the overall autologous regeneration capacity is limited due to a limited blood supply as well as an unfavorable surgical approach. In this context, currently cell-based strategies, implantable scaffolds and specific bioactive molecules dominate the efforts in the field of tissue engineering [18].

First described by Friedenstein et al., mesenchymal stromal cells (MSC) represent a highly investigated population given their unique biological properties [19,20,21]. Based on the heterogeneity of isolation and cultivation procedures in different laboratories, the International Society for Cellular Therapy (ISCT) defined criteria for identifying unique populations of MSCs [22]. However, these criteria do not fully support the purification of homogeneous MSC populations. Indeed, isolation of MSCs according to ISCT criteria results in heterogeneous, non-clonal cultures of stromal cells containing stromal cells with different multipotent properties, committed progenitor cells, and differentiated cells as well. Subsequently, it has been scientifically accepted that MSC represent a heterogeneous population containing a subgroup of stem cells [23]. Mesenchymal stromal cells derived from adipose tissue (adipose-derived stromal cells, ASC) show similar properties to those of bone marrow-derived MSCs, but have about 500 x the amount of MSCs per gram of adipose tissue [24]. Although the fatty tissue can easily be processed by collagenase after harvesting, a heterogeneous cell fraction, the stromal vascular fraction (SVF), is formed first. It contains granulocytes, monocytes, lymphocytes, endothelial cells, pericytes, erythrocytes and other cells in addition to a variable amount of stromal cells [25]. In a previous study of our research group, existing purification strategies were modified by adding an additional plastically adherent short-term incubation after SVF isolation. It was shown that the heterogeneity of the resulting cell fraction was significantly reduced, comparing to SVF [26]. As an ASC containing and GMP-compliant cell product (DE_BB_01_GMP_2017_1018), *Oxacells HP* was ready-to-use in less than 24 h. The isolated cells were able to differentiate into adipocytes, chondrocytes and osteoblasts and expressed proteins previously associated with the beneficial effects of MSCs. The aim of the present animal study was a comparative analysis of the chondroregenerative potential of intra-articular Oxacells HP therapy in temporomandibular joint osteoarthritis.

## 2. Results

The surgical procedure could be performed in all animals without complications. In the follow up period, all animals showed stage-appropriate wound healing without clinical evidence of a local inflammatory reaction or wound dehiscence. At the beginning of the study the rabbits showed a normal distribution regarding size and weight, on whose basis (without explicit measurements) also a normal distribution of the temporomandibular joint size was assumed. We did not observe any adverse immune reactions from the use of xenogeneic human MSC in fully immunocompetent rabbits.

### 2.1. Histological Analysis and Cartilage Thickness Measurement

**Figure 1:**
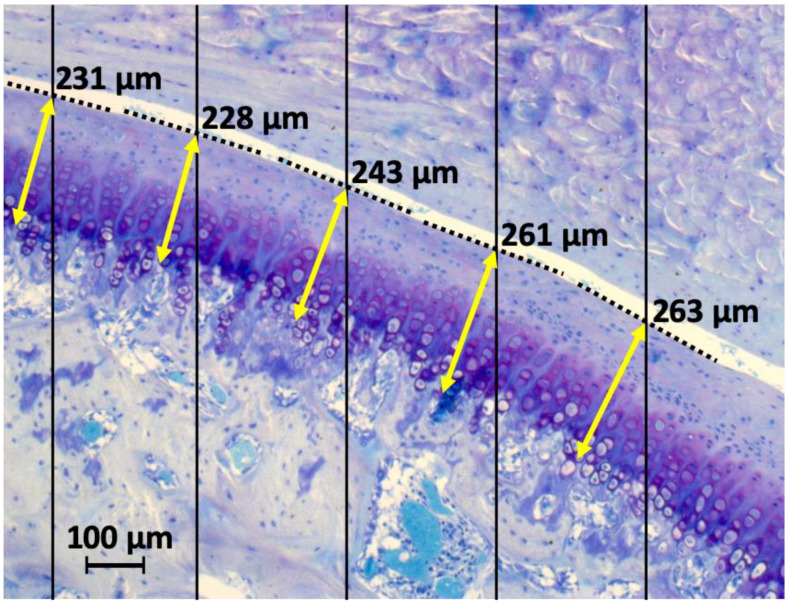
To objectify the cartilage thickness measurements, a line grid (black) was used to define random measuring points on the cartilage surface. At these points the thickness measurement (yellow) was finally taken, each right angled to an imaginary tangent (dotted).

Histological analysis of mandibular condyles was performed in all groups of all animals (temporomandibular joints). In the stromal cell-treated groups, more chondral tissue seems to integrate into the marginal zones of the cartilage defect. Mostly a cluster formation instead of a columnar arrangement was observed. Subchondral bone sclerosis or disruption of the osteochondral junction was not observed in any group. Histological examination of the cartilage showed a significant increase in cartilage thickness in the stromal cell-treated groups (Table 1). The measurements were performed at five points determined by a random grid (Figure 1).

**Figure 2:**
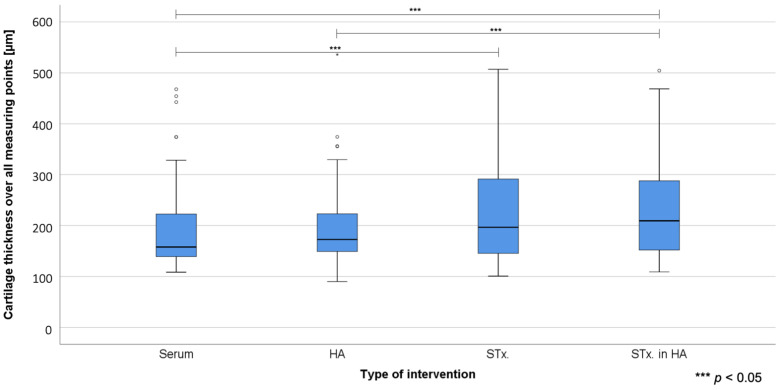
Boxplot analysis showing the underlying distribution of final cartilage thickness vs. the four treatment groups. Circles represent outliers with more than 1.5 times interquartile range. Significant group differences are marked (***).

**Figure 3:**
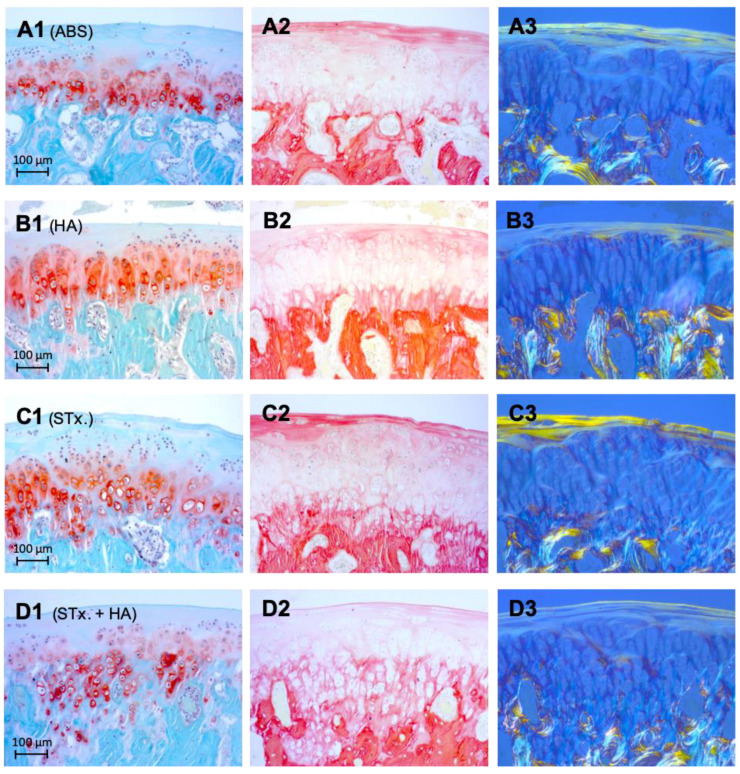
(**A**–**D**). Histological analysis 4 weeks after intra-articular injection therapy (1 = Safranin-O, 2 = Picrosirius red, 3 = Picrosirius red (polarized)). (**A1**–**A3**) representing group 1 (ABS), (**B1**–**B3**) group 2 (HA), (**C1**–**C3**) group 3 (STx.), and (**D1**–**D3**) group 4 (STx. + HA). Collagen I on cartilage surface could be visualized much better in group 3 (STx.) than in the other groups (Picrosirius red: red; Picrosirius red (polarized): yellow-orange), indicating a comparatively higher cartilage regeneration. A statistical significance analysis was not performed. Overall, no significant increased or decreased GAG accumulation could be detected in any of the groups using Safranin-O staining.

Figure 2 provides the boxplot analysis of cartilage thickness measurements. Significant differences were found between STx. + HA (group 4) vs. Serum (group 1) (*p* = 0.028); STx. + HA (group 4) vs. HA (group 2) (*p* = 0.042) and STx. (group 3) vs. Serum (group 1) (*p* = 0.036). The difference between STx. (group 3) and HA (group 2) with a *p* = 0.054 does not quite reach the required significance level. Figure 3 shows histological analysis using Safranin-O and Picrosirius red staining.

### 2.2. Back-Scattered Electron Imaging (BEI)

**Figure 4:**
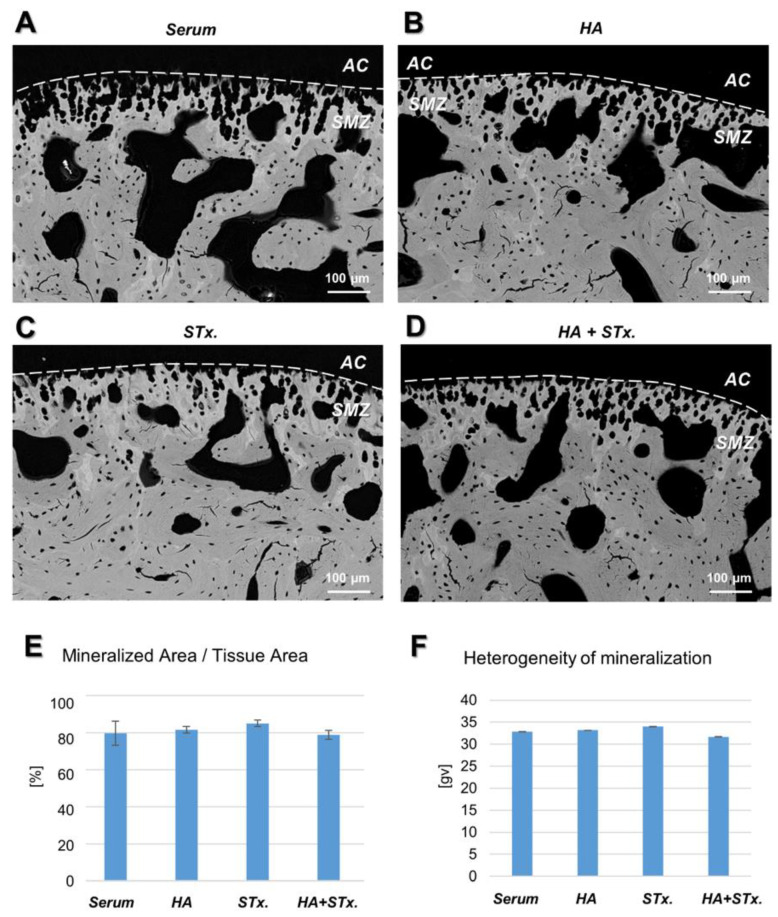
Representative back-scattered images of temporomandibular joint (TMJ) specimens of all groups. (**A**–**D**) The images show a cross-sectional view of the TMJ, whereby darker pixels correspond to a low degree of mineralization and brighter pixels correspond to higher degree of mineralization. The articular cartilage (AC) is located on top of the subchondral mineralized zone (SMZ) which is composed of calcified cartilage containing chondrocyte lacunae and subchondral bone containing osteocyte lacunae. (**E**) In the calcified cartilage zone, the ratio of mineralized area per tissue area, an inverse measure of tissue porosity, was similar between the groups. (**F**) The heterogeneity of mineralization, assessed as the full-width-half-maximum of the gray value histogram, was similar between groups.

Results of the back-scattered electron microscopy imaging performed of the TMJ are displayed in Figure 3. Black pixels at the top of the images represent non-mineralized regions of articular cartilage (AC). This is followed by the subchondral mineralized zone (SMZ), composed of calcified cartilage, containing chondrocyte lacunae, and subchondral bone, containing osteocyte lacunae (Figure 4A–D). As indicated in Figure 4E, the ratio of mineralized area to tissue area of the subchondral mineralized zone was similar in all groups (*p* = 0.404). The heterogeneity of mineralization is visualized in terms of gray values in each image, whereby darker pixels represent lower mineralized regions, and brighter pixels represent higher mineralized regions. Figure 4F indicates a similar heterogeneity of gray values in the calcified cartilage zone in all groups (*p* = 0.166).

## 3. Discussion

This study aimed to investigate the effect of local stromal cell therapy as part of a regenerative approach for the treatment of osteoarthritis of the temporomandibular joint in an animal model. In this context, the use of adipose tissue derived stromal cells resulted in a significantly increased cartilage thickness compared to the control groups, whereby the additional use of hyaluronic acid seemed to further increase this effect. Results of the back-scattered electron microscopy imaging of the subchondral mineralized zone revealed no significant treatment effects in terms of tissue porosity and heterogeneity of mineralization. In an in-vitro preliminary study we could show that existing purification strategies can be modified by adding an additional plastically adherent short-term incubation after SVF isolation in order to significantly reduce the heterogeneity of the resulting cell fraction compared to SVF. Furthermore, a chondrogenic, adipogenic and osteogenic differentiation potential of the isolated stromal cells could be demonstrated [26].

Regeneration techniques of the temporomandibular joint must match the anatomical, architectural and functional characteristics of the entire temporomandibular joint unit, including the mandibular condyle, articular cup, articular disc and ligaments. In this regard, temporomandibular joint cartilage differs significantly from other hyaline joint cartilages in terms of histology and structure. For example, as opposed to most synovial joints, the articular surfaces of the temporomandibular joint are not covered by hyaline cartilage, but by a layer of fibrous tissue (containing abundant type I collagen and proportionally less type II collagen) [27]. In addition, TMJ condylar fibrocartilage contains fewer glycosaminoglycans (GAGs) than other hyaline joint cartilage [28]. Furthermore, the subcellular (including GAGs) and cellular (including fibroblasts, fibrocytes and fibrochondrocytes) composition of the temporomandibular joint disc differs from the hyaline cartilage of the joint surfaces [29,30].

The limitations of current therapeutics for TMJ-OA have led to an increased interest in regenerative strategies, which are primarily fed by knowledge gained from regenerative orthopaedic medicine. In addition to restoring function, regenerative strategies are also measured by the avoidance of ossified or fibrous adhesions, which are one of the main complications of artificial TMJ replacement [31]. Besides implantable scaffolds and specific bioactive molecules, cell-based strategies dominate the efforts in the field of tissue engineering. In this context, stromal cells are of special interest because of their multilineage plasticity, with potential induction toward both fibrocartilage and articular cartilage as well as osseous tissue and structures of the ligamentary system.

Several studies have shown an increased volume of meniscus fibrocartilage including regenerative capacity associated with stem/stromal cell treatment, starting in the conventional orthopaedic indications [32,33]. Depending on their tissue origin, mesenchymal stroma cells have been proven to show significant differences in their differentiation capacity into specific cell lines [34]. Although the use of MSCs for the treatment of osteoarthritis has gained both scientific and clinical relevance in recent years, there still is a lack of research on its use in the temporomandibular joint. Especially the ability of adipose tissue-derived stromal cells (ADSC) to achieve sufficient differentiation and proliferation even in an oxygen-deficient environment seems to appropriately address the limited vascularization of the temporomandibular joint [35]. However, to the best of the authors’ knowledge, valid clinical thickness measurements in a prospective randomized controlled trial in the temporomandibular joint as in our study are not yet available. Recently, various experimental and clinical approaches inducing repair and regeneration in the TMJ based on cell therapy have been reported [36,37].

Carboni et al. analyzed the effect of the injection of fat-derived stromal cells in the temporomandibular joint as a new treatment option for TMD [38]. Eight patients were randomized into two groups. The patients in the study group were treated with arthrocentesis and autologous fat derived stromal cell injection (stromal vascular fraction, single application). The patients in the control group were treated with saline injection only. Although osteoarthritis was not the primary focus of this study (even being one of the exclusion criteria), the MRI results showed an impressive restoration of the anatomy of the structures involved compared to the control group, with almost complete restoration of the morphology of the discus and capsular-ligamentous structures. In addition to ADSCs, various tooth-derived stromal cells (periodontal ligament stromal cells (PDLSCs), stromal cells from apical papilla (SCAPs), dental follicle progenitor cells (DFPCs) and dental pulp stromal cells (DPSCs)), also demonstrate the chondrogenic and osteogenic differentiation capacity so crucial for potential TMJ regeneration processes [39,40,41]. Wu et al. investigated the impact of TMJ-derived synovial MSCs on temporomandibular disc repair in a small animal model (immunodeficient mice) [21]. In their pilot study synovial MSCs from the temporomandibular joint were cultured in a fibrin-chitosan composite scaffold with TGF-β3 and the corresponding cell-scaffold-construct was subsequently implanted into the punched temporomandibular disc explants and transplanted into the dorsum of the animals in vivo. Four weeks after implantation, a substantial fibrocartilage formation with characteristic deposition of type I and II collagen was observed [21].

Wang et al. first investigated the clinical effect of xenogeneic human adipose-derived mesenchymal progenitor cells (MPCs) on knee joint osteoarthritis in rabbits [42]. To induce moderate to severe OA conditions, an anterior cruciate ligament transaction (ACLT) was performed in combination with a complete medial meniscectomy. The research group found that the eroded cartilage was almost completely covered by the repaired tissues and cartilage thickness increased significantly from 324 μm in the hyaluron group (control) to 400 μm in the haMPC group. With regard to the increase in cartilage thickness, there is a clear consistency with our study results. In addition to the chondral effects, potential subchondral changes are also of interest. In this regard, our results suggest that the recovering effects of stromal cells as induced in the articular cartilage four weeks post single injection, were not associated with a simultaneous recovery of the subchondral hard tissue. This observation is in line with a recent study on arthritic knee joints of rats, where subchondral bone mineral density was not changed after 6 and 12 weeks post-intra-articular delivery of HA alone nor combined with MSCs [43]. The reasons for missing subchondral effects are certainly multifactorial, conceivable are, a too short interval between therapy and examination as well as an insufficient effect of a single treatment for subchondral effects. In their study on stem cell-mediated effects on knee joint OA, Wang et al. did not investigate potential subchondral effects [42].

In a recent study in New Zealand white male rabbits, Kim et al. demonstrated the therapeutic efficacy of human umbilical cord matrix-mesenchymal stromal cells hUCM-MSC for the treatment of TMJ-OA and also identified a concentration and cell line dependent efficacy [44]. In addition, it was observed that the anti-inflammatory effects were similar to those of dexamethasone injections, but cartilage regeneration effects were only observed in the MSC-treated group. These results suggest that the isolated use of anti-inflammatory injections has significant limitations in the treatment of TMJ-OA. Zhang et al. investigated the role of MSC exosomes in the regulation of nociceptive behaviour, inflammatory response, and condylar cartilage and subchondral bone healing in an immunocompetent rat model of temporomandibular joint osteoarthritis [45]. This research was based on the assumption that paracrine secretion of trophic factors (in particular exosomes) plays a major role for the regenerative effects of many MSC-based therapies [46,47]. In their study in an immunocompetent rat model, they demonstrated that MSC exosomes mediate repair and regeneration of the osteoarthritic TMJs through early reduction in inflammation to suppress pain and tissue degeneration followed by increased proliferation and matrix synthesis to restore the TMJ osteochondral tissues 8 weeks after intra-articular injection [45]. With regard to a cartilage thickness measurement also performed, Zhang et al. described a lower fibrous cartilage thickening in the exosome-treated group compared to the control group after 4 and 8 weeks. This difference to the results of our study and its interpretation was explained by possible subchondral bone erosion, with an overall reduction in condyle height observed in the OA+ phosphate-buffered saline (PBS) group despite increased fibrocartilage thickening. In contrast to cartilage thickness, our own back-scattered electron imaging (BEI) analysis showed no significant differences in subchondral structures (although condylar height was not explicitly measured), so there is finally a difference between the results and their interpretation. However, several studies have shown that the exosomes only have very short half-lives of 2-5 min in the blood and therefore require repeated injections [47].

Searching for an explanation regarding the best results in our study the group HA + STx. rapidly leads to the discussion about the use of scaffolds and carriers to optimize MSC transplantation [48,49]. Hyaluronic acid (HA) is an important component of the synovial fluid protecting the articular cartilage by lubricating and absorbing shocks. From clinical research it is known that during osteoarthritis the HA concentration decreases, which aggravates the damage to the cartilage. For several animal models of articular cartilage damage, the efficacy of MSCs combined with HA has been found to be superior to those of MSCs or HA alone [50,51]. This is in line with our research findings for the temporomandibular joint. Li et al. demonstrated a superior therapeutic effect of mesenchymal bone marrow stromal cells BMSCs in combination with HA for neocartilage formation in the knee joint in a beagle-dog model against HA alone and against saline [52]. Chiang et al. also reported a significant reduction in osteoarthritis progression after intra-articular injection of allogeneic MSCs suspended in hyaluronic acid (HA) compared to isolated injection treatment with HA in rabbits (knee) [53]. Using acrylated hyaluronic acid as a scaffold for bone morphogenic protein-2 (BMP-2) and human mesenchymal stromal cells (hMSCs) for rat calvarial defect regeneration, Kim et al. found that the hydrogels with BMP-2 and MSCs had the highest expression of osteocalcin and mature bone formation with vascular markers, such as CD31 and vascular endothelial growth factors, in comparison to control groups [54]. The HA coating the articular cartilage surface is located near the collagen fibrils and sulfated proteoglycans in cartilage and has been identified as a crucial modulator in many physiological and pathological processes in cartilage [55]. HA not only serves as a vehicle for MSCs delivery, but also has potential biological effects, including promotion of synovial cell or chondrocyte migration processes as well as enhancement of chondrogenic effects of MSCs [56,57]. However, the optimal ratio of HA to MSCs for engraftment remains to be clarified.

In this context, besides hyaluronic acid PRP also seems to be a promising medium for an improved differentiation capacity and finally efficacy of local stem cell therapy. Wittig et al. demonstrated that a PRP clot represents an excellent three-dimensional scaffold for MSC in which these cells can proliferate, differentiate and migrate [58]. Exploring the ability of MSC to induce cartilage regeneration in the temporomandibular joint, the same research group was able to show extensive cartilaginous regeneration after transplantation of PRP-embedded human MSC in a murine animal model [59]. In contrast to our study, the initial iatrogenic cartilage damage was performed alone surgically. The time of animal sacrifice and temporomandibular joint removal with subsequent macroscopic and microscopic examination was, at six weeks after initiation of therapy, a total of two weeks behind our design. The results were primarily descriptive without measuring cartilage thickness as a clinically relevant endpoint similar to our study.

Overall, our results suggest that intra-articular injection of a stem cell-containing, GMP-compliant ATMP can support articular regeneration in TMJ-OA. However, the study presented here has several limitations. It is first necessary to distinguish between defined stem/stromal cell products and the Oxacells HP as a stem cell containing, GMP-compliant ATMP used here. In contrast to cultured ASCs and in line with the initial hypothesis in our preliminary work [26], Oxacells HP still express CD34+ cells and not yet CD105. Regarding this, little is known about how CD34 may affect biological features and the functionality of SVF cells and/or ASCs. Maumus et al. showed that CD34+ ASCs are the only subpopulation of ASC containing clonogenic cells, and the only one able to differentiate into adipogenic and osteogenic lineages [60]. It must be noted that Oxacells HP is still less homogenous than cultivated ASCs and the surface marker expression did not reach the criteria of cultivated ASCs as defined by the International Federation for Adipose Therapeutics (IFATS) and Science and the International Society for Cellular Therapy (ISCT). However, the homogeneity, multipotency and paracrine activity of Oxacells HP was sufficiently qualified as an ATMP and the described manufacturing process was authorized by the national regulatory agencies (DE_BB_01_GMP_2017_1018).

There are further methodological limitations beyond this. Rabbits show a different chewing-functional load of the TMJ than humans. Furthermore, it should be noted that the combined chemical and mechanical induction of a cartilage defect does not adequately reflect the degenerative environment of chronic osteoarthritis of the temporomandibular joint. A group of untreated, arthritis-induced-only rabbits could also have extended the analysis and data interpretation. Due to the complex involvement of all internal joint structures, future studies should also be expanded to include investigations of the synovial membrane, subchondral bone, joint capsule and synovial fluid. Moreover, long-term stability data could be supplemented by prolonged multi-stage analysis and thus be included in clinical and practical considerations regarding dosage and application regimes.

## 4. Materials and Methods

The study was designed as a randomized, controlled experimental study. It conforms to the Guide for the Care and Use of Laboratory Animals, eighth edition, updated by the U.S. National Research Council Committee in 2011 and was performed in accordance with the European Directive 2010/63 EU. The experimental design was officially reviewed, and animal ethics approval was obtained from the Federal Office of Consumer Protection, Food Safety and Veterinary Affairs (7 Nov 2016, Reference Nr. 74/16) meeting the German and European Animal Welfare guidelines.

### 4.1. Animals—General Information/Anesthesia and Surgery

This animal study was performed at the Laboratory Animal Facility of the University Medical Center Hamburg (University Medical Center, Hamburg, Germany). The protocol assigned 28 female New Zealand White Rabbits (average weight 800 g, age approximately 12 weeks) to four treatment groups. All animals were held under standardized conditions in single cages according to the German law for animal experiments. All animals underwent surgery and were sacrificed after 8 weeks for further detailed evaluation of the joint area. Prior to surgery, animals received 5 mg/kg Xylazin (Rompun^®^, Bayer, Leverkusen, Germany) + 50 mg Ketamin (Ursotamin^®^, Serumwerk Bernburg, Germany) subcutaneously. Additionally, the rabbits received an antibiosis (enrofloxacin 10 mg/kg body weight). The fur was shaved over the joint area and desinfected. Anesthesia was maintained by subsequent dosing via an intravenous cannula via the ear vein with Ketamine/Xylazine.

In all rabbits, 50 μL of 4 mg/mL collagenase solution (Clostridium histolyticum type II, 425 units/mL enzyme activity) was injected intra-articularly into both temporomandibular joints, according to the methodology described by Huh et al. adapted for the temporomandibular joint [61]. In addition, a blood sample and a computed tomography (CT Scan, Timepoint T0) were performed for further examination. Four weeks (T1) after the stimulus, CT-graphic confirmation of the development of osteoarthritis was obtained, followed by intra-articular injection treatment (both sides) according to randomization. Group 1: AB Serum (ABS), 150 µL; Group 2: Hyaluronic acid (HA), 150 µL; Group 3: Mesenchymal stromal cells (STx.), 1 × 10^6^ MSC/mL in 150 µL saline; Group 4: Mesenchymal stromal cells in hyaluronic acid (HA + STx.), 1 × 10^6^ MSC/mL in 150 µL HA. The hyaluronic acid applied, GO-ON (Rottapharm|Madaus, Monza, Italy), is a preparation of sodium hyaluronate obtained by fermentation from Streptococcus equi, with an intermediate molecular weight of 800,000–1,500,000 Dalton, available in 2.5 mL prefilled syringes and a concentration of 10 mg/mL. In group 4, the final Oxacells HP preparation was mixed with the hyaluronic acid (GO-ON). After diluting the hyaluronic acid with 0.9% sodium chloride (B. Braun, Melsungen, Germany) in a 1:10 ratio to realize mixability (corresponding to a final HA target concentration of 1mg/mL), mixing with Oxacells HP was performed approximately 4 h before application in a 1:1 ratio. The cell solution together with the HA was adjusted to a final concentration of 1 × 10^6^ MSC/mL, corresponding to 150,000 cells in 150 µL. Postoperative analgesia was performed by subcutaneous application of 0.03 mg/kg Temgesic. All animals postoperatively were checked daily. After a total of 8 weeks, the animals were euthanized and the jaws including the temporomandibular joint were removed. The manufacturing process of the stromal cell product Oxacells HP (Oxacell^®^ AG, Potsdam) used has been described in detail within our preliminary work [26].

### 4.2. Harvesting, Processing and Characterization of Stromal Cells (Preliminary Work)

The comprehensive process of creating a stem cell containing GMP-compliant advanced therapy medicinal product (ATMP), named Oxacells HP, as well as its further detailed characterization was carried out as part of a preliminary study by our research group, which was published in 2019 [26]. In order to provide a complete scientific traceability, the most important aspects are briefly summarized here.

#### 4.2.1. SVF Isolation and Short-Term Incubation

Samples of subcutaneous lipoaspirate (300–3300 mL) were obtained from patients undergoing cosmetic liposuction by power-assisted liposuction (PAL) as described before by Barzelay et al. [62]. After further processing as described by Born et al. [26], the cells were seeded on cell culture dishes (Greiner Bio-One) at high density (≥1.0 × 105 cells/cm^2^) in DMEM-F12, 2% KnockOut™ SR XenoFree Medium (ThermoFisher Scientific, Waltham, MA, USA) and incubated for 16–20 h (37 °C, 6% CO_2_, 95% RH) for short-term incubation and purification. Subsequently, the cells were trypsinized (Trypsin-EDTA solution, Sigma-Aldrich, Munich, Germany) and suspended in PBS w/o Mg^2+^ Ca^2+^ (Biochrom, Berlin, Germany), 2% KnockOut™ (ThermoFisher Scientific, Waltham, USA). After the washing step, the cells were filtered through a cell strainer (Steriflip^®^, Merck Millipore, Darmstadt, Germany), washed two more times with 0.9% sodium chloride (B. Braun, Melsungen, Germany) and suspended in a buffered solution for further analysis. The entire procedure from lipoaspirate to finished cell product took approximately 24 h. The manufacturing protocol was GMP certified (DE_BB_01_GMP_2017_1018) according to Art. 13 and 15 of Directive 2001/20/EC. The resulting short-term incubated cell fraction was called Oxacells HP (OXACELL^®^ AG, Potsdam, Germany).

The resulting cells were randomly divided into two groups, one of which was directly used for further experiments (Oxacells HP), whereas the cells of the second group were again seeded on cell culture dishes (2000–4000 cells/cm^2^) and expanded with DMEM-F12 (ThermoFisher Scientific, Waltham, USA) with 10% (w/v) pooled human serum (GMP-grade, Center for Clinical Transfusion Medicine, Tübingen, Germany) until confluence and criteria for ASCs were reached (8–14 days, corresponding to passage 0) [63].

#### 4.2.2. Immunophenotypic Characterization of Short-Term Incubated Cells (Oxacells HP) In Vitro

The following antibodies coupled to fluorescein isothiocyanate (FITC), phycoerythrin (PE), or allophycocyanin (APC) were used for immunophenotypic characterization of short-term incubated cells (Oxacells HP): Anti-CD-13, anti-CD31, anti-CD34, anti-CD44, anti-CD45, anti-CD73, anti-CD90, anti-CD105-FITC, anti-CD235a, and anti-HLA-DR, DP, and DQ (Miltenyi Biotec, Bergisch Gladbach, Germany).

As described by Born et al., immunophenotypic characterization of Oxacells HP was performed with fixed gates and protocols according to the GMP guideline, implemented as part of the preliminary study, and published accordingly [26]. According to Bourin et al., CD13 (Alanine aminopeptidase), CD44, CD73 (5’-ribonucleotide phosphohydrolase), CD90 (Thy-1), and CD105 (Endoglin) were selected as positive surface markers for ASCs whereas CD31 (PECAM-1), CD45 (leukocyte common antigen), and CD235a (glycophorin A) were selected as ASC negative markers [63]. Additionally, the unstable positive marker CD344 was measured. The analysis showed a significant increase of all stable positive markers (CD13, CD44, CD73, CD90, and CD105) from the first purification to Oxacells HP and further to the cultivated ASCs. The stromal/stem cell content (CD13+, CD44+, CD73+, and CD90+) was significantly higher in the Oxacells HP fraction than in the purification steps before, whereas the number of other cells then ASCs (CD31+, CD45+, CD235a+ and HLA II+ in Figure 3B) was significantly reduced in the Oxacells HP, indicating that the Oxacells HP population is significantly less heterogeneous than the upstream intermediate products.

#### 4.2.3. Multilineage Differentiation of Short-Term Incubated Cells (Oxacells HP) In Vitro

The differentiation tests were done according to Zhu et al., using human serum from pooled human male AB plasma (Sigma-Aldrich, Munich, Germany) instead of animal-derived FBS for all experiments [64]. All differentiation experiments were compared to undifferentiated controls. To check the stromal/stem cell characteristics of the ASCs in the short-term incubated cell fraction, the cells were seeded on chamber slides and differentiated into adipogenic, chondrogenic, and osteogenic lineages. In contrast to the undifferentiated controls, the differentiated cells could be stained with the appropriate dyes according to their lineage (Oil Red O, Alcian Blue, and Kossa), indicating a successful differentiation capacity [26].

### 4.3. Histological Analysis

The study group consisted of 28 rabbits (7 per group) and accordingly 56 temporomandibular joints. The samples were embedded in polymethylmethacrylate (PMMA) as described by Hahn et al. previously to allow artifact-free undecalcified preparation of bone and cartilage tissue while preserving the matrix and cellular features [65]. Prior to embedding, the samples were first fixed in 4% phosphate-buffered formaldehyde and then dehydrated in an ascending ethanol series (70%, 90%, 100% ethanol). Afterwards, the bone specimens were embedded without decalcification in PMMA according to the instructions provided by the manufacturer (Merck, Darmstadt, Germany). After the completion of the polymerization, the embedded suture samples had a cylindrical block shape, which is appropriate for microtome cutting. Two microtome-cut sections with a thickness of approximately 4 µm were stained according to standard procedures per case. The resulting histological sections were stained according to Masson Goldner, by Kossa van Gieson and with toluidine blue. For cartilage thickness measurements over a total of five random measurement points, a line grid was generated, and the cartilage thickness was measured rectangular to an imaginary tangent.

### 4.4. Scanning Electron Microscopy

Back-scattered electron imaging (BEI) was performed to assess the morphology, porosity and heterogeneity of mineralization within the subchondral mineralized zone of the temporomandibular joint. A total of eight MMA blocks were polished coplanar and coated with carbon. The left and right jaw of one animal per group were imaged in a scanning electron microscope (Crossbeam 340, Zeiss, Oberkochen) at 20 kV, a working distance of 20 mm and 300× magnification. All imaging parameters were kept constant during the imaging process. The resulting images were thresholded based on gray values in order to distinguish between mineralized tissue (brighter pixels), and pores (dark pixels) using ImageJ [66]. The curves in the gray value histograms of each image were evaluated for the full-width-half maximum as a measure for heterogeneity of mineralization.

### 4.5. Statistical Analysis

For the statistical evaluation, the rabbits were first presented in a descriptive way. For the analysis of the primary question a single factor analysis of variance (ANOVA) was chosen. The necessary assumptions were tested and no relevant deviations could be detected. Within the analysis p-values with *p* < 0.05 are considered to be statistically significant. The testing was performed in a hierarchical order according to the closed multiple test procedure, whereby the *p*-values were adjusted on global and local levels for a multiple test.

Group comparisons of electron microscopy-derived data were performed using a Oneway ANOVA for normally distributed data (mineralized area/tissue area) and Kruskal-Wallis-Test for non-normally distributed data (width of gray value histogram) using SPSS (IBM SPSS Statistics 22).

## 5. Conclusions

The single intra-articular injection of a stem cell containing, GMP-compliant advanced therapy medicinal product for the treatment of iatrogenically induced osteoarthritis of the temporomandibular joint demonstrated a significantly increased chondroregenerative potential compared to the control groups. These results suggest that intra-articular stromal/stem cells may be an effective treatment option for osteoarthritis of the temporomandibular joint. However, further studies are needed to ensure a complete transfer of these results to human temporomandibular osteoarthritis and to implement stem cell treatment into clinical routine, including dose and therapy regimen setting.

## Figures and Tables

**Table 1:** Overview of group-specific cartilage thickness measurements (across all measurement points), showing mean values and standard deviations.

Group	Cells/Fluid	Mean (M, µm)	Standard Deviation (SD, µm)
1	AB Serum (ABS)	195.26	86.78
2	Hyaluronic Acid (HA)	195.24	81.58
3	Mesenchymal Stromal Cells (STx.)	228.78	103.62
4	Mesenchymal Stromal Cells in hyaluronic acid (HA + STx.)	231.14	92.52

## Data Availability

Data is contained within the article or supplementary material.

## Abbreviations

ATMP	Advanced therapy medicinal product
TMJ-OA	Temporomandibular joint osteoarthritis
TMJ	Temporomandibular joint
TMD	Temporomandibular disorder
ABS	Treatment group 1: AB Serum
HA	Treatment group 2: Hyaluronic acid
STx.	Treatment group 3: Mesenchymal stromal cells (Oxacells HP)
HA + STx.	Treatment group 4: Hyaluronic acid + Mesenchymal stromal cells (Oxacells HP)
TMJD	Temporomandibular joint disorders
RDC	Research Diagnostic Criteria
TENS	Transcutaneous electrical nerve stimulation
NSAID	Non-steroidal anti-inflammatory drugs
PRP	Platelet Rich Plasma
MSC	Mesenchymal stromal cells
ESC	Embryonic stromal cells
ISCT	International Society for Cellular Therapy
ASC	Adipose-derived stromal cells
SVF	Stromal vascular fraction
BEI	Back-scattered electron imaging
AC	Articular cartilage
SMZ	Subchondral mineralized zone
GAG	Glycosaminoglycans
ADSC	Adipose tissue-derived stromal cells
PDLSC	Periodontal ligament stromal cells
SCAP	Stromal cells from apical papilla
DFPC	Dental follicle progenitor cells
DPSC	Dental pulp stromal cells
MPC	Mesenchymal progenitor cells
hMSC	Human mesenchymal stromal cells
ACLT	Anterior cruciate ligament transaction
PBS	Phosphate-buffered saline
PMMA	Polymethylmethacrylate

## References

[1] Gopal K., Shankar R., Vardhan H. (2014). Prevalence of temporo‑mandibular joint disorders in symptomatic and asymptomatic patients: A cross‑sectional study. Int. J. Adv. Sci..

[2] Milam S. (2003). Pathophysiology and epidemiology of TMJ. J. Musculoskelet. Neuronal Interact..

[3] Su N., Liu Y., Yang X., Shen J., Wang H. (2018). Association of malocclusion, self-reported bruxism and chewing-side preference with oral health-related quality of life in patients with temporomandibular joint osteoarthritis. Int. Dent. J..

[4] Dworkin S., LeResche L. (1992). Research diagnostic criteria for temporomandibular disorders: Review, criteria, examinations and specifications, critique. J. Craniomandib. Disord..

[5] Wang X.D., Zhang J.N., Gan Y.H., Zhou Y.H. (2015). Current Understanding of Pathogenesis and Treatment of TMJ Osteoarthritis. J. Dent. Res..

[6] Alqutaibi A., Aboalrejal A. (2015). Types of occlusal splint in management of temporomandibular disorders (TMD). J. Arthritis.

[7] Ok S.-M., Lee J., Kim Y.-I., Lee J.-Y., Kim K.B., Jeong S.-H. (2014). Anterior condylar remodeling observed in stabilization splint therapy for temporomandibular joint osteoarthritis. Oral Surg. Oral Med. Oral Pathol. Oral Radiol..

[8] Nicolakis P., Erdogmus C.B., Kollmitzer J., Kerschan-Schindl K., Sengstbratl M., Nuhr M., Crevenna R., Fialka-Moser V. (2002). Long-term outcome after treatment of temporomandibular joint osteoarthritis with exercise and manual therapy. Cranio.

[9] Treacy K. (1999). Awareness/relaxation training and transcutaneous electrical neural stimulation in the treatment of bruxism. J. Oral Rehabil..

[10] Cairns B.E. (2010). Pathophysiology of TMD pain–basic mechanisms and their implications for pharmacotherapy. J. Oral Rehabil..

[11] Hersh E.V., Balasubramaniam R., Pinto A. (2008). Pharmacologic management of temporomandibular disorders. Oral Maxillofac. Surg. Clin. N. Am..

[12] Guarda-Nardini L., Stifano M., Brombin C., Salmaso L., Manfredini D. (2007). A one-year case series of arthrocentesis with hyaluronic acid injections for temporomandibular joint osteoarthritis. Oral Surg. Oral Med. Oral Pathol. Oral Radiol..

[13] Hegab A.F., Ali H.E., Elmasry M., Khallaf M.G. (2015). Platelet-rich plasma injection as an effective treatment for temporomandibular joint osteoarthritis. J. Oral Maxillofac. Surg..

[14] Manfredini D., Bonnini S., Arboretti R., Guarda-Nardini L. (2009). Temporomandibular joint osteoarthritis: An open label trial of 76 patients treated with arthrocentesis plus hyaluronic acid injections. Int. J. Oral Maxillofac. Surg..

[15] Rigon M., Pereira L.M., Bortoluzzi M.C., Loguercio A.D., Ramos A.L., Cardoso J.R. (2011). Arthroscopy for temporomandibular disorders. Cochrane Database Syst. Rev..

[16] Thambar S., Kulkarni S., Armstrong S., Nikolarakos D. (2020). Botulinum toxin in the management of temporomandibular disorders: A systematic review. Br. J. Oral Maxillofac. Surg..

[17] Guarda-Nardini L., Manfredini D., Ferronato G. (2008). Temporomandibular joint total replacement prosthesis: Current knowledge and considerations for the future. Int. J. Oral Maxillofac. Surg..

[18] Van Bellinghen X., Idoux-Gillet Y., Pugliano M., Strub M., Bornert F., Clauss F., Schwinté P., Keller L., Benkirane-Jessel N., Kuchler-Bopp S. (2018). Temporomandibular joint regenerative medicine. Int. J. Mol. Sci..

[19] Friedenstein A.J., Petrakova K.V., Kurolesova A.I., Frolova G.P. (1968). Heterotopic transplants of bone marrow. Transplantation.

[20] Takahashi K., Tanabe K., Ohnuki M., Narita M., Ichisaka T., Tomoda K., Yamanaka S. (2007). Induction of pluripotent stem cells from adult human fibroblasts by defined factors. Cell.

[21] Wu Y., Gong Z., Li J., Meng Q., Fang W., Long X. (2014). The pilot study of fibrin with temporomandibular joint derived synovial stem cells in repairing TMJ disc perforation. BioMed Res. Int..

[22] Dominici M., Le Blanc K., Mueller I., Slaper-Cortenbach I., Marini F., Krause D., Deans R., Keating A., Prockop D., Horwitz E. (2006). Minimal criteria for defining multipotent mesenchymal stromal cells. The International Society for Cellular Therapy position statement. Cytotherapy.

[23] Squillaro T., Peluso G., Galderisi U. (2016). Clinical trials with mesenchymal stem cells: An update. Cell Transplant..

[24] Fraser J.K., Wulur I., Alfonso Z., Hedrick M.H. (2006). Fat tissue: An underappreciated source of stem cells for biotechnology. Trends Biotechnol..

[25] Zuk P.A., Zhu M., Mizuno H., Huang J., Futrell J.W., Katz A.J., Benhaim P., Lorenz H.P., Hedrick M.H. (2001). Multilineage cells from human adipose tissue: Implications for cell-based therapies. Tissue Eng..

[26] Born S., Dörfel M.J., Hartjen P., Yekani S.A.H., Luecke J., Meutsch J.K., Westphal J.K., Birkelbach M., Köhnke R., Smeets R. (2019). A short-term plastic adherence incubation of the stromal vascular fraction leads to a predictable GMP-compliant cell-product. Bioimpacts.

[27] Griffin C., Hawthorn R., Harris R. (1975). Anatomy and histology of the human temporomandibular joint. Monogr. Oral Sci..

[28] Delatte M., Von den Hoff J.W., Van Rheden R.E., Kuijpers-Jagtman A.M. (2004). Primary and secondary cartilages of the neonatal rat: The femoral head and the mandibular condyle. Eur. J. Oral Sci..

[29] Detamore M.S., Hegde J.N., Wagle R.R., Almarza A.J., Montufar-Solis D., Duke P.J., Athanasiou K.A. (2006). Cell type and distribution in the porcine temporomandibular joint disc. J. Oral Maxillofac. Surg..

[30] Plumb M., Aspden R.M. (2005). The response of elderly human articular cartilage to mechanical stimuli in vitro. Osteoarthr. Cartil..

[31] Mehrotra D. (2013). TMJ bioengineering: A review. J. Oral Biol. Craniofac. Res..

[32] Centeno C.J., Busse D., Kisiday J., Keohan C., Freeman M., Karli D. (2008). Increased knee cartilage volume in degenerative joint disease using percutaneously implanted, autologous mesenchymal stem cells. Pain Physician.

[33] Vangsness Jr C.T., Jack Farr I., Boyd J., Dellaero D.T., Mills C.R., LeRoux-Williams M. (2014). Adult human mesenchymal stem cells delivered via intra-articular injection to the knee following partial medial meniscectomy: A randomized, double-blind, controlled study. J. Bone Joint Surg. Am..

[34] Pizzute T., Lynch K., Pei M. (2015). Impact of tissue-specific stem cells on lineage-specific differentiation: A focus on the musculoskeletal system. Stem Cell Rev..

[35] Mäenpää K., Ellä V., Mauno J., Kellomäki M., Suuronen R., Ylikomi T., Miettinen S. (2010). Use of adipose stem cells and polylactide discs for tissue engineering of the temporomandibular joint disc. J. R. Soc. Interface.

[36] Zhang S., Yap A.U., Toh W.S. (2015). Stem cells for temporomandibular joint repair and regeneration. Stem Cell Rev..

[37] Ahtiainen K., Mauno J., Ellä V., Hagström J., Lindqvist C., Miettinen S., Ylikomi T., Kellomäki M., Seppänen R. (2013). Autologous adipose stem cells and polylactide discs in the replacement of the rabbit temporomandibular joint disc. J. R. Soc. Interface.

[38] Carboni A., Amodeo G., Perugini M., Arangio P., Orsini R., Scopelliti D. (2019). Temporomandibular disorders clinical and anatomical outcomes after fat-derived stem cells injection. J. Craniofacial Surg..

[39] Saito M.T., Silvério K.G., Casati M.Z., Sallum E.A., Nociti Jr F.H. (2015). Tooth-derived stem cells: Update and perspectives. World J. Stem Cells.

[40] Guo L., Li J., Qiao X., Yu M., Tang W., Wang H., Guo W., Tian W. (2013). Comparison of odontogenic differentiation of human dental follicle cells and human dental papilla cells. PLoS ONE.

[41] Sedgley C.M., Botero T.M. (2012). Dental stem cells and their sources. Dent. Clin. N. Am..

[42] Wang W., He N., Feng C., Liu V., Zhang L., Wang F., He J., Zhu T., Wang S., Qiao W. (2015). Human adipose-derived mesenchymal progenitor cells engraft into rabbit articular cartilage. Int. J. Mol. Sci..

[43] Xing D., Wu J., Wang B., Liu W., Liu W., Zhao Y., Wang L., Li J.J., Liu A., Zhou Q. (2020). Intra-articular delivery of umbilical cord-derived mesenchymal stem cells temporarily retard the progression of osteoarthritis in a rat model. Int. J. Rheum. Dis..

[44] Kim H., Yang G., Park J., Choi J., Kang E., Lee B.-K. (2019). Therapeutic effect of mesenchymal stem cells derived from human umbilical cord in rabbit temporomandibular joint model of osteoarthritis. Sci. Rep..

[45] Zhang S., Teo K.Y.W., Chuah S.J., Lai R.C., Lim S.K., Toh W.S. (2019). MSC exosomes alleviate temporomandibular joint osteoarthritis by attenuating inflammation and restoring matrix homeostasis. Biomaterials.

[46] Toh W.S., Foldager C.B., Pei M., Hui J.H.P. (2014). Advances in mesenchymal stem cell-based strategies for cartilage repair and regeneration. Stem Cell Rev..

[47] Zhang S., Chu W., Lai R., Lim S., Hui J., Toh W. (2016). Exosomes derived from human embryonic mesenchymal stem cells promote osteochondral regeneration. Osteoarthr. Cartil..

[48] Li Y., Cheng H., Cheung K., Chan D., Chan B. (2014). Mesenchymal stem cell-collagen microspheres for articular cartilage repair: Cell density and differentiation status. Acta Biomater..

[49] Ferreira M.S.V., Jahnen-Dechent W., Labude N., Bovi M., Hieronymus T., Zenke M., Schneider R.K., Neurs S. (2012). Cord blood-hematopoietic stem cell expansion in 3D fibrin scaffolds with stromal support. Biomaterials.

[50] Suhaeb A.M., Naveen S., Mansor A., Kamarul T. (2011). Hyaluronic acid with or without bone marrow derived-mesenchymal stem cells improves osteoarthritic knee changes in rat model: A preliminary report. Indian J. Exp. Biol..

[51] Sato M., Uchida K., Nakajima H., Miyazaki T., Guerrero A.R., Watanabe S., Roberts S., Baba H. (2012). Direct transplantation of mesenchymal stem cells into the knee joints of Hartley strain guinea pigs with spontaneous osteoarthritis. Arthritis Res. Ther..

[52] Li L., Duan X., Fan Z., Chen L., Xing F., Xu Z., Chen Q., Xiang Z. (2018). Mesenchymal stem cells in combination with hyaluronic acid for articular cartilage defects. Sci. Rep..

[53] Chiang E.-R., Ma H.-L., Wang J.-P., Liu C.-L., Chen T.-H., Hung S.-C. (2016). Allogeneic mesenchymal stem cells in combination with hyaluronic acid for the treatment of osteoarthritis in rabbits. PLoS ONE.

[54] Kim J., Kim I.S., Cho T.H., Lee K.B., Hwang S.J., Tae G., Noh I., Lee S.H., Park Y., Sun K. (2007). Bone regeneration using hyaluronic acid-based hydrogel with bone morphogenic protein-2 and human mesenchymal stem cells. Biomaterials.

[55] Yoshioka M., Shimizu C., Harwood F.L., Coutts R.D., Amiel D. (1997). The effects of hyaluronan during the development of osteoarthritis. Osteoarthr. Cartil..

[56] Kavalkovich K.W., Boynton R.E., Murphy J.M., Barry F. (2002). Chondrogenic differentiation of human mesenchymal stem cells within an alginate layer culture system. In Vitro Cell. Dev. Biol. Anim..

[57] Maniwa S., Ochi M., Motomura T., Nishikori T., Chen J., Naora H. (2001). Effects of hyaluronic acid and basic fibroblast growth factor on motility of chondrocytes and synovial cells in culture. Acta Orthop. Scand..

[58] Wittig O., Diaz-Solano D., Cardier J. (2018). Viability and functionality of mesenchymal stromal cells loaded on collagen microspheres and incorporated into plasma clots for orthopaedic application: Effect of storage conditions. Injury.

[59] Gomez M., Wittig O., Diaz-Solano D., Cardier J.E. (2020). Mesenchymal Stromal Cell Transplantation Induces Regeneration of Large and Full-Thickness Cartilage Defect of the Temporomandibular Joint. Cartilage.

[60] Maumus M., Peyrafitte J.-A., d’Angelo R., Fournier-Wirth C., Bouloumié A., Casteilla L., Sengenès C., Bourin P. (2011). Native human adipose stromal cells: Localization, morphology and phenotype. Int. J. Obes..

[61] Huh J.-E., Park Y.-C., Seo B.-K., Lee J.-D., Baek Y.-H., Choi D.-Y., Park D.-S. (2013). Cartilage protective and chondrogenic capacity of WIN-34B, a new herbal agent, in the collagenase-induced osteoarthritis rabbit model and in progenitor cells from subchondral bone. Evid. Based Complementary Altern. Med..

[62] Barzelay A., Levy R., Kohn E., Sella M., Shani N., Meilik B., Entin-Meer M., Gur E., Loewenstein A., Barak A. (2015). Power-assisted liposuction versus tissue resection for the isolation of adipose tissue–derived mesenchymal stem cells: Phenotype, senescence, and multipotency at advanced passages. Aesthet. Surg. J..

[63] Bourin P., Bunnell B.A., Casteilla L., Dominici M., Katz A.J., March K.L., Redl H., Rubin J.P., Yoshimura K., Gimble J.M. (2013). Stromal cells from the adipose tissue-derived stromal vascular fraction and culture expanded adipose tissue-derived stromal/stem cells: A joint statement of the International Federation for Adipose Therapeutics and Science (IFATS) and the International Society for Cellular Therapy (ISCT). Cytotherapy.

[64] Zhu M., Heydarkhan-Hagvall S., Hedrick M., Benhaim P., Zuk P. (2013). Manual isolation of adipose-derived stem cells from human lipoaspirates. J. Vis. Exp..

[65] Hahn M., Vogel M., Delling G. (1991). Undecalcified preparation of bone tissue: Report of technical experience and development of new methods. Virchows Arch..

[66] Schneider C.A., Rasband W.S., Eliceiri K.W. (2012). NIH Image to ImageJ: 25 years of image analysis. Nat. Methods.

